# Wind speed and soil properties drive the height-diameter allometric pattern of island plants

**DOI:** 10.3389/fpls.2025.1548664

**Published:** 2025-03-26

**Authors:** Chengfeng Yang, Renfu Liao, Shengzhuo Huang, Yikang Cheng, Shurong Zhou

**Affiliations:** ^1^ Key Laboratory of Genetics and Germplasm Innovation of Tropical Special Forest Trees and Ornamental Plants, Ministry of Education, School of Tropical Agriculture and Forestry, Hainan University, Haikou, China; ^2^ Hainan Key Laboratory for Research and Development of Natural Products from Li Folk Medicine, Institute of Tropical Bioscience and Biotechnology, Chinese Academy of Agricultural Sciences, Haikou, China; ^3^ School of Ecology, Hainan University, Haikou, China

**Keywords:** height-diameter allometry, island plants, island area, soil, wind speed, temperature

## Abstract

**Introduction:**

Island ecosystems, due to their geographical isolation and unique environmental conditions, often serve as natural laboratories for ecological research and are also sensitive to global climate change and biodiversity loss. The allometric relationship between plant height-diameter reflects the adaptive growth strategy of plants under different environmental conditions, particularly in response to biomechanical constraints (e.g., wind resistance) and resource availability. This study aims to explore the key driving factors of the height-diameter allometry of island plants, focusing on how island area, soil properties, and climatic factors (e.g., wind speed, temperature, and precipitation) affect plant growth strategy.

**Methods:**

We analyzed plant data from 20 tropical islands, using SMA regression to calculate the allometric exponent and intercept for each island’s plants, and evaluated the effects of island area, soil properties, and climatic factors (wind speed, temperature, and precipitation) on the height-diameter allometric relationship.

**Results:**

The results show that island area has no significant effect on plant allometry, while climatic factors, particularly wind speed, and soil properties significantly influence the allometric exponent and intercept, respectively. Specifically, wind speed is the primary driver of the height-diameter allometric exponent, regulating plant growth proportions through mechanical stress and canopy limitation. In contrast, soil properties predominantly govern changes in the allometric intercept, reflecting their critical role in determining baseline growth conditions, such as resource allocation and initial morphological adaptation. The effects of temperature and precipitation are relatively weak, likely due to the buffering effects of the tropical climate and marine moisture supplementation.

**Discussion:**

Overall, this study highlights the key roles of wind speed and soil in shaping the allometry of island plants, providing new insights into the adaptive strategies of island plants under resource limitations and climatic pressures, as well as offering important scientific evidence for island ecological conservation and restoration.

## 1 Introduction

Allometric relationships, particularly between height and diameter, are crucial in plant growth and ecological adaptation (King, 1996; Chave et al., 2005). They reflect how plants optimize their morphological structure to cope with environmental pressures (e.g., wind, temperature) and resource limitations, thereby enhancing their competitive ability and ecological adaptability through adaptive growth strategies (Chave et al., 2005, 2014; Thomas et al., 2015; Xiao et al., 2021). Island ecosystems are one of the hotspots of biodiversity loss, and the growth patterns of insular plants are more susceptible to environmental changes due to the unique environmental and geographical conditions. Thus, there is an urgent need for a thorough study of island ecosystems in the context of global change and increasing human disturbance (MacArthur and Wilson, 1967; Whittaker et al., 2017; Fernández-Palacios et al., 2021; Manes et al., 2021). Indeed, plant growth strategies vary among species and environments, and these strategies can influence allometric relationships, thereby helping plants maximize their survival and reproductive capacities under limited resources (West et al., 1999; Thomas et al., 2015). Therefore, studying the height-diameter allometric relationship of island plants is of great significance for predicting how plants respond to environmental changes, as well as guiding the conservation and restoration of island ecosystems (Tilman, 1988; Whittaker et al., 2017).

**Figure 1:**
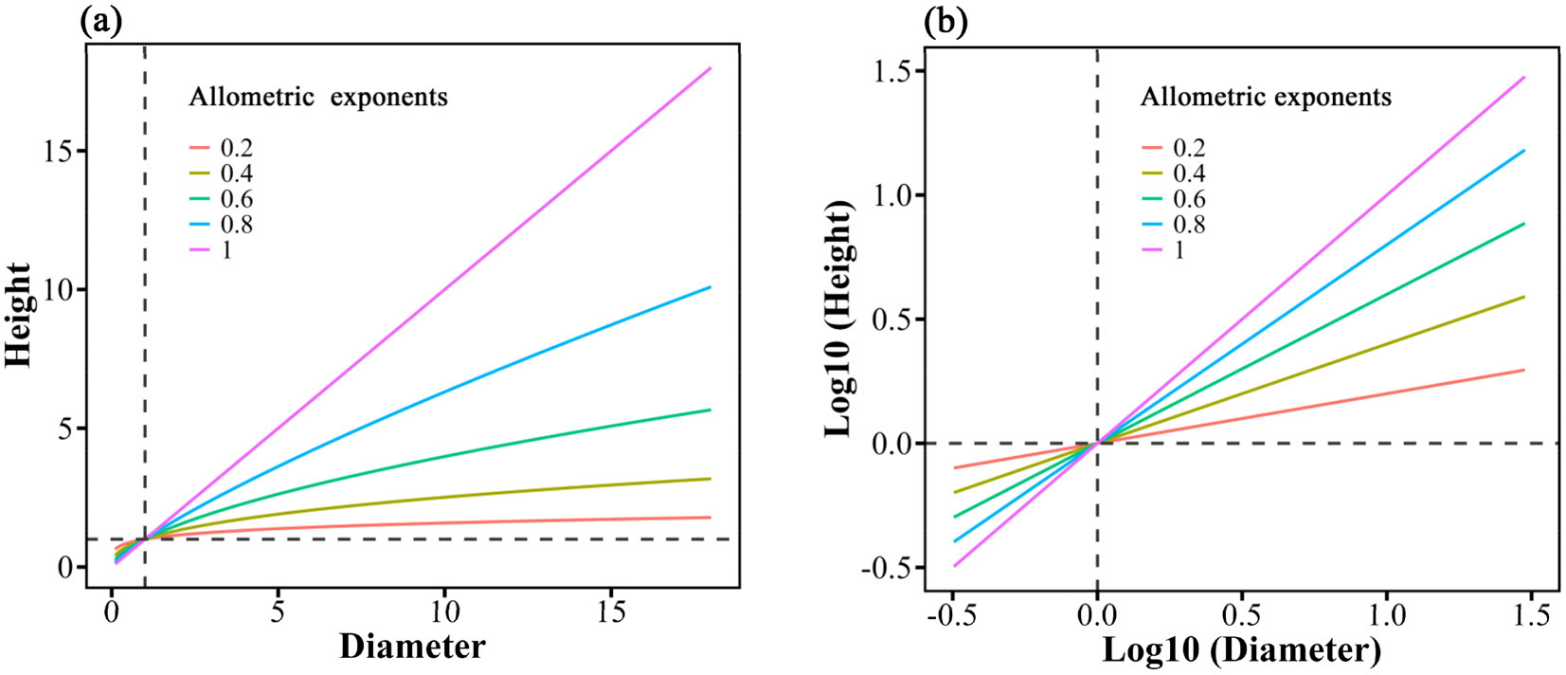
Schematic of allometry for different heights and diameters, illustrating the power-law expression **(a)** and its log10-transformed expression **(b)**. Lines of different colors represent different height-diameter allometric exponent **(b)** values. a: H = *aD^b^*. b: Log (H) = log (*a*) + *b* log (D).

Height and diameter are core indicators of plant growth, playing key roles in resource acquisition and structural stability (Tilman, 1988; Poorter et al., 2012). Specifically, plant height directly affects the ability to capture light resources and is a primary determinant of carbon sequestration capacity (Moles et al., 2009), while diameter relates to the mechanical support and water transport efficiency (Niklas, 1993; Vizcaíno-Palomar et al., 2016). The dynamic balance between these height and diameter determines the plant growth pattern and performance (Chave et al., 2005; Qiu et al., 2021). The allometric relationship between height and diameter is typically expressed in the power function form: *H=a D^b^*, where H is the tree height, *D* is the diameter of a cross-section of a tree trunk 1.3 meters above the ground (i.e., DBH) for trees, or the basal diameter for forbs; *a* is the constant coefficient, or intercept meaning the height when *D* =1, and *b* is the height-diameter allometric exponent (West et al., 1999; Brown et al., 2004). When the allometric exponent *b*=1, height and diameter grow at the same rate, the plant height and diameter increase proportionally. When *b*<1, the growth rate of height is lower than that of diameter, indicating a greater emphasis on enhancing the supporting structure (**Figure 1a**). The height-diameter allometric relationship reflects plant adaptation strategies under different environmental conditions, with plants tending to increase diameter under resource limitation and height under resource availability. Xiao et al. (2021) found that nitrogen addition affects plant allometry, subsequently altering species diversity and community structure, highlighting the close relationship between allometric relationship, resource use efficiency, and competitive ability. Additionally, the logarithmic form of the function is often used to analyze the relationship between height and diameter: log (H) = log (*a*) + *b* log (D), this form facilitates a clearer understanding of the height-diameter allometric pattern through regression analysis (Thomas et al., 2015; Xiao et al., 2021) (**Figure 1b**). In this equation, *b* still represents the height-diameter allometric exponent, and *a* is the intercept, which reflects the initial proportional relationship between the two variables (i.e., height and diameter) in the allometric equation. It reveals the differences in the growth starting points or baseline levels of plants under varying environmental conditions (Feldpausch et al., 2011).

Mechanistically, many theories suggest that the allometric relationship of plants would be influenced by various biological factors such as metabolism, biomechanical constraints, and hydraulic transport, making the relationship generally predictable. For example, *b* = 3/2 is often cited (McMahon, 1973; West et al., 1997; Brown et al., 2004). However, more evidence now shows that the allometric relationship exhibits a certain degree of plasticity, with abiotic factors such as climate conditions, soil properties, and geographical traits, potentially overriding the predictability of biological factors, thereby exerting a strong influence on allometry (Hulshof et al., 2015; Thomas et al., 2015; Zhang et al., 2020; Xiao et al., 2021). For instance, a previous study has shown that the height of all trees in Spain was shorter than predicted by biomechanical models (Lines et al., 2012). Due to the unique environmental conditions, such as resource limitation, soil infertility, and extreme climate variations, this could lead to more significant changes in height-diameter allometric relationships (Hulshof et al., 2015; Thomas et al., 2015). Therefore, island ecosystems are an excellent place to explore the potential driving mechanisms of height-diameter allometric relationships.

Island area is one of the most important features of island ecosystems, and the positive relationship between island size and species richness has been widely confirmed (MacArthur and Wilson, 1967; Zhou et al., 2024). With further research, many studies have found that island area not only influences species richness (Whittaker et al., 2017; Ottaviani et al., 2020), but also exerts significant effects on various ecological processes, such as community structure, species interactions, and the process of species succession across multiple trophic levels (Patiño et al., 2017; Zhou et al., 2024). Thus, island size may be a potential factor affecting the plant growth relationships. However, whether and how the island area influences the plant height-diameter allometric relationship remains unclear.

Furthermore, soil properties and nutrient availability are critical for plant height growth, which may, in turn, affect height-diameter growth relationships. Indeed, nutrient-rich soils could promote vertical growth in plants (Givnish et al., 2014), while nutrient-poor soils may drive plants to alter their growth strategies (Cramer, 2012), subsequently altering their allometric relationships (Xiao et al., 2021; Xu et al., 2022). On the other hand, many studies have been conducted on the effects of climate factors (e.g., temperature and precipitation) on plant allometry (Olson et al., 2018; Fortin et al., 2019; Liu et al., 2021). For example, there is considerable variation in the height-diameter allometric relationships of trees across America, and temperature and precipitation have been confirmed as the main contributing factors (Hulshof et al., 2015). Likewise, temperature has been found to be the primary factor influencing the tree height-diameter relationship in the southern and northeastern regions of China (Wang et al., 2006; Zhang et al., 2019). Further, Liu et al. (2021) also found that tree height was mainly positively influenced by precipitation in Arizona. In island ecosystems, particularly in tropical regions, wind speed not only differs significantly from that on mainland, but islands are also more prone to tropical storms (Young et al., 2011; Jiang et al., 2022). Indeed, high wind speeds and strong biomechanical disturbances may affect plant morphology and survival strategies, such as the formation of bent tree shapes, deepening of root systems, or horizontal growth (King, 1986; Meng et al., 2006; Niklas and Spatz, 2006; Jackson et al., 2021). A study on Dominica Island in the West Indies, one of the regions with the highest wind speeds in the tropical zone, found that tree heights were 30-116% shorter than those in other tropical regions, and the height-diameter allometric relationship showed a distinct depression compared to other areas (Thomas et al., 2015). Nevertheless, there is limited research on the impact of climate conditions, especially wind speed, on plant height-diameter allometric relationship in island ecosystems (Watt and Kirschbaum, 2011; Thomas et al., 2015; Young et al., 2011).

Many studies have also shown that extreme climate factors (such as precipitation in the wettest month, temperature in the warmest month, and wind speed in the windiest month) often have a more significant impact on plant growth than average climate indicators. Intense environmental stress or more favorable conditions are more likely to lead to changes in plant growth patterns in terms of tree height and diameter (Cunningham and Read, 2002; Frank et al., 2015; Rudley et al., 2023). For example, a global-scale study on plant height found that precipitation in the wettest month was the best explanatory factor for plant height variation (Moles et al., 2009). Moreover, maximum wind speed and extreme events such as tropical storms have a strong impact on plant height and survival (Thomas et al., 2015; Bauman et al., 2022). Given the intensifying global climate change, understanding the influence of these extreme environmental variables on plant growth mechanisms has become especially important. Therefore, we conducted research in the South China Sea region, investigating the height-diameter allometric relationships of plants surveyed on twenty islands. The study aims to address the following questions: 1) What is the relationship between island area, soil properties, various climate variables, and the height-diameter allometric relationship of island plants? 2) What are the key factors influencing the height-diameter allometric relationship of plants on islands in the South China Sea region?

## 2 Materials and methods

### 2.1 Study site and sampling method

As an important tropical marine ecosystem in China, the South China Sea comprises a rich array of islands, coral reefs, and shallow marine areas, showcasing diverse marine environments and abundant biodiversity. The South China Sea not only provides favorable conditions for the survival of numerous marine species but also serves as a habitat and breeding ground for tropical and subtropical plants. The plant communities on the islands in this region exhibit significant variations due to island area, soil type, and climatic conditions (Zhou et al., 2024). Despite more than 50 islands occurring within this region, considering factors such as whether the island is suitable for investigation (with a certain area to allow for sampling plots, and suitable woody plant communities rather than just a few herbaceous species) and whether it is easy to climb the islands, we ultimately selected the 20 most representative islands with a size varies from 1ha to 400ha for detailed surveys, which were conducted from April 2023 to May 2024.

For each island, we established different numbers of permanent 20m × 20m sampling plots, after logarithmically transforming the area of the islands we planned to survey, we adjusted the number of plots based on the actual situation, ensuring that the number of plots on each island is proportional to the transformed island area. To minimize spatial autocorrelation effects, we ensured that the distance between any two sampling plots on the same island exceeded 100 meters. Additionally, the vegetation status and habitat differences of the island were considered as supplementary information when selecting the sampling plots. For the smallest islands, where the limited area prevented the establishment of plots more than 100 meters apart, we maintained a minimum distance of 40 meters between plots. In total, we established 87 sampling plots on 20 islands in this region.

Within these plots, we conducted plant surveys and soil sampling. The plot setup was based on RTK (Real-Time Kinematic) positioning provided by Xunwei Positioning, which allowed for more precise positioning compared to traditional methods and avoided the influence of slope on the projected area of the plots. RTK was used to locate the four corners of each plot, and rubber tubing and red plastic rope were used to mark and fix the boundaries of the plots. Within each plot, we inventoried all woody plants rooted within the plots and recorded plant height and diameter, Plant height was measured using a height pole, with the height recorded by aligning the top of the canopy with the marked scale on the pole. Stem diameter was measured at breast height (1.3 m above the ground) using a diameter tape. Species were identified on-site using morphological methods, and further identification was done with reference to the Flora of Chinese (2019). Soil samples were collected using a diagonal sampling method. Three 2m × 2 m subplots were evenly distributed along the diagonal of each plot, and four soil samples (10 cm in depth) were randomly taken from each subplot. Then, all samples were combined and homogenized within a plot into one composite sample. In total, 261 soil samples were collected.

### 2.2 Data acquisition

In this study, we used a logarithmic regression model to calculate the height-diameter allometric relationship of plants, which describes the growth relationship between plant height and diameter. Specifically, we used the standard major axis (SMA) regression to estimate the height-diameter allometric exponent and intercept for the plants surveyed on each island. SMA regression is preferred for allometric studies as it accounts for measurement error in both the independent (diameter) and dependent (height) variables. To facilitate subsequent analysis, we calculated the height-diameter allometric exponent (*b*) and intercept (*a*) for each plant species surveyed across the islands. The logarithmic linear model used in this study is as follows:


log (H)=a+b log (D)

Where H represents plant height (in meters), D represents plant diameter (in centimeters), *a* is the intercept of the regression equation, reflecting the initial proportional relationship between height and diameter, *b* is the height-diameter allometric exponent, which represents the scaling relationship between diameter and height. For the SMA regression, we used the sma() function from the smatr package in R with the argument robust = T, which provides robust estimates of the slope and intercept, minimizing the influence of outliers. This method allows us to derive the strength and trend of the relationship between diameter and height for each plant species on different islands, ensuring more accurate allometric calculations.

We measured five soil physicochemical properties that may influence plant communities, with the specific measurement methods as follows: Soil pH was measured using the Metter-S210 SevenCompact pH analyzer, with a soil-to-water ratio of 1:2.5; Soil organic carbon (SOC) content was determined by the K2Cr2O7 redox titration method; Total nitrogen (TN) content was measured using the semi-micro Kjeldahl method; Total phosphorus (TP) concentration was determined by colorimetric analysis using a UV-Visible spectrophotometer; Total potassium (TK) content was extracted with 1 M ammonium acetate and analyzed by inductively coupled plasma technology.

Climate data were sourced from the WorldClim Global Climate and Weather Data (Fick and Hijmans, 2017). We downloaded bioclimatic variables including temperature and precipitation data, as well as wind speed datasets. Subsequently, we used the “***terra***” package to process the downloaded TIF files. To explore the impact of extreme climate factors on plant height-diameter allometric relationship, we extracted the annual maximum temperature of the hottest month (hereafter temperature), the annual precipitation of the wettest month (hereafter precipitation), and the annual wind speed of the windiest month from the dataset (hereafter wind).

### 2.3 Statistical analysis

Before analysis, we applied Principal Component Analysis (PCA) to the five soil variables (SOC, N, P, K, pH) to simplify the data structure (Abdi and Williams, 2010; Ren et al., 2022). We retained the first PCA axis as the integrated predictor for each group of variables, as the first principal component explained the largest percentage of variance in soil properties (PC_soil_).

Then we used linear regression to analyze the effects of island characteristics (i.e., area), environmental conditions (i.e., PCsoil), and climatic factors (i.e., wind speed, temperature, and precipitation) on plant height-diameter allometric relationship. We treated the allometric exponent *b* and intercept *a* as dependent variables, with abiotic factors as independent variables, to investigate the relationship between island environmental conditions and plant height-diameter allometric exponent and intercept. This approach aimed to explain the link between plant height-diameter allometric relationship and island conditions.

To investigate the relative importance of island area, soil properties, and climate factors on the height-diameter allometric exponent and intercept of plant height-diameter relationships, we estimated the relative importance of each variable using the “glmm.hp” function (Lai et al., 2022). All variables were standardized using the “scale” function. All statistical analyses were performed in R v.4.4.1 (R Core Team, 2013).

## 3 Results

### 3.1 The effects of island area and soil factors on island plant allometric relationship

**Figure 2:**
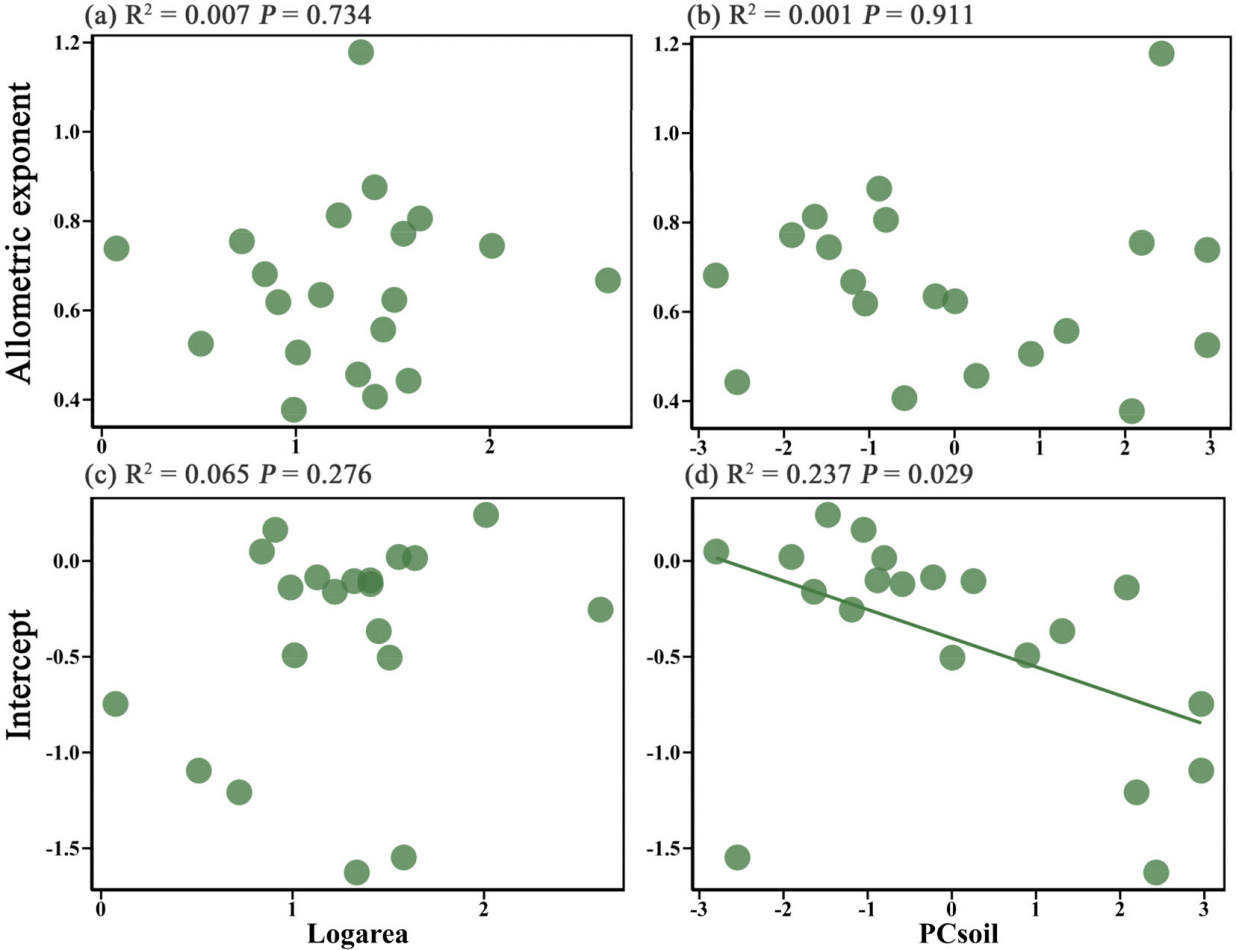
Effect of island area on the height-diameter allometric exponent **(a)** and intercept **(c)** of island plants, and the effect of soil properties (PC_soil_) on the height-diameter allometric exponent **(b)** and intercept **(d)** of island plants.

Linear regression analysis revealed that island area had no significant effect on either the allometric exponent or intercept (*P* > 0.05) (**Figures 2a, c**). Likewise, soil factors also exerted an insignificant effect on the allometric exponent (*P* > 0.05) (**Figure 2b**), while their influence on the intercept approached significance (*P* = 0.029) (**Figure 2d**). This result suggests that soil properties may influence the plant height-diameter allometric relationship by regulating the intercept.

### 3.2 The effects of climate factors on island plant allometric relationship

**Figure 3:**
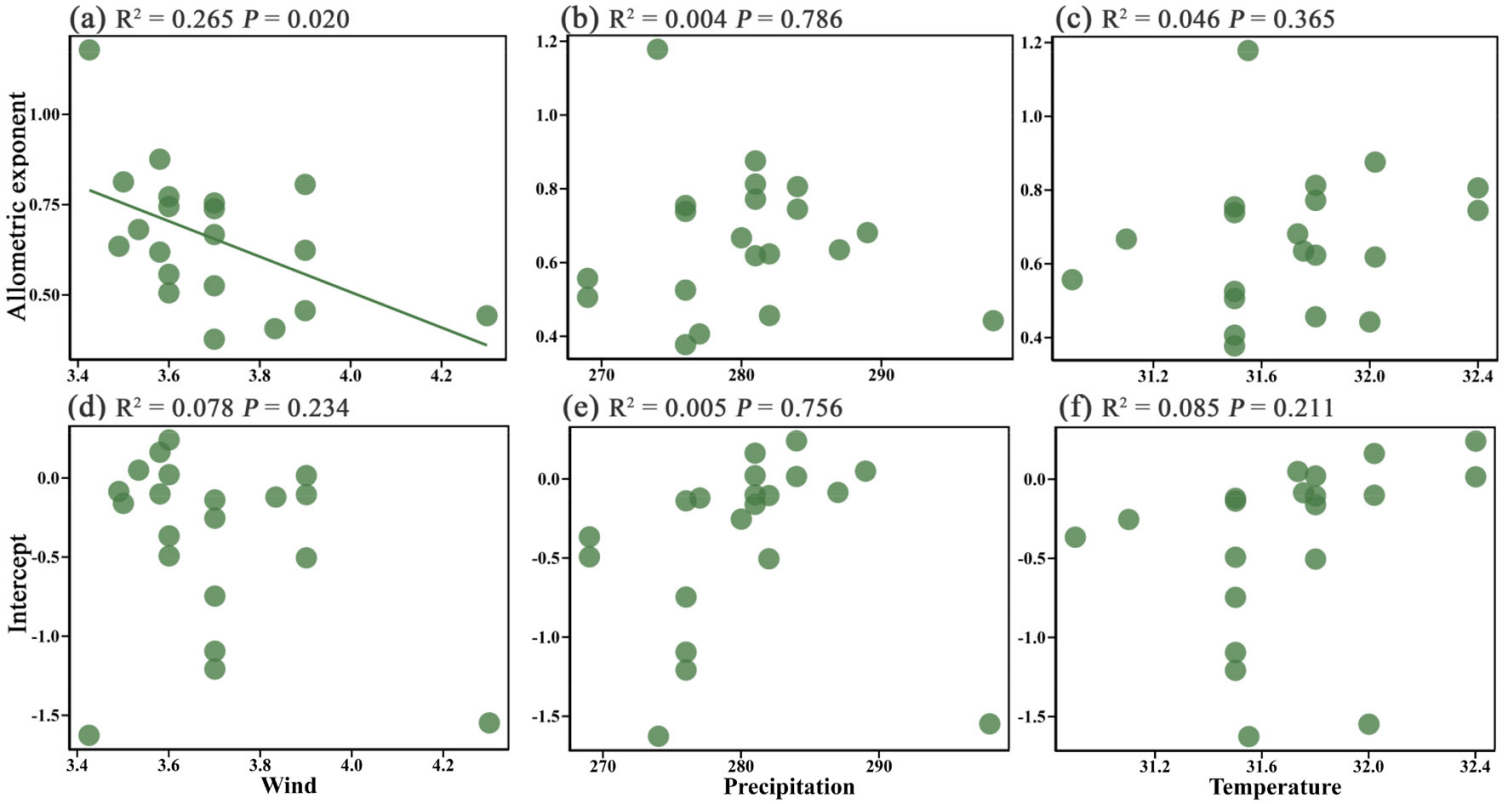
Effect of wind speed **(a, d)**, precipitation **(b, e)**, and temperature **(c, f)** on the height-diameter allometric exponent and intercept of island plants respectively.

Regression analysis results showed that wind speed, precipitation, and temperature had different effects on the height-diameter allometric exponent and intercept of plants. Specifically, wind speed had a significant effect on the height-diameter allometric exponent (*P* = 0.02) but no significant effect on the intercept (*P* > 0.05) (**Figures 3a, d**). As wind speed increased, the height-diameter allometric exponent decreased, indicating a clear suppressive effect of wind speed on plant growth patterns. In contrast, temperature and precipitation showed no significant effects on either the height-diameter allometric exponent or intercept (*P* > 0.05) (**Figures 3b, c, e, f**). This suggests that wind speed is the primary factor influencing the allometric relationship of island plants in the study area, while the effects of precipitation and temperature are relatively limited.

### 3.3 Relative importance of different factors affecting island plant allometric relationship

**Figure 4:**
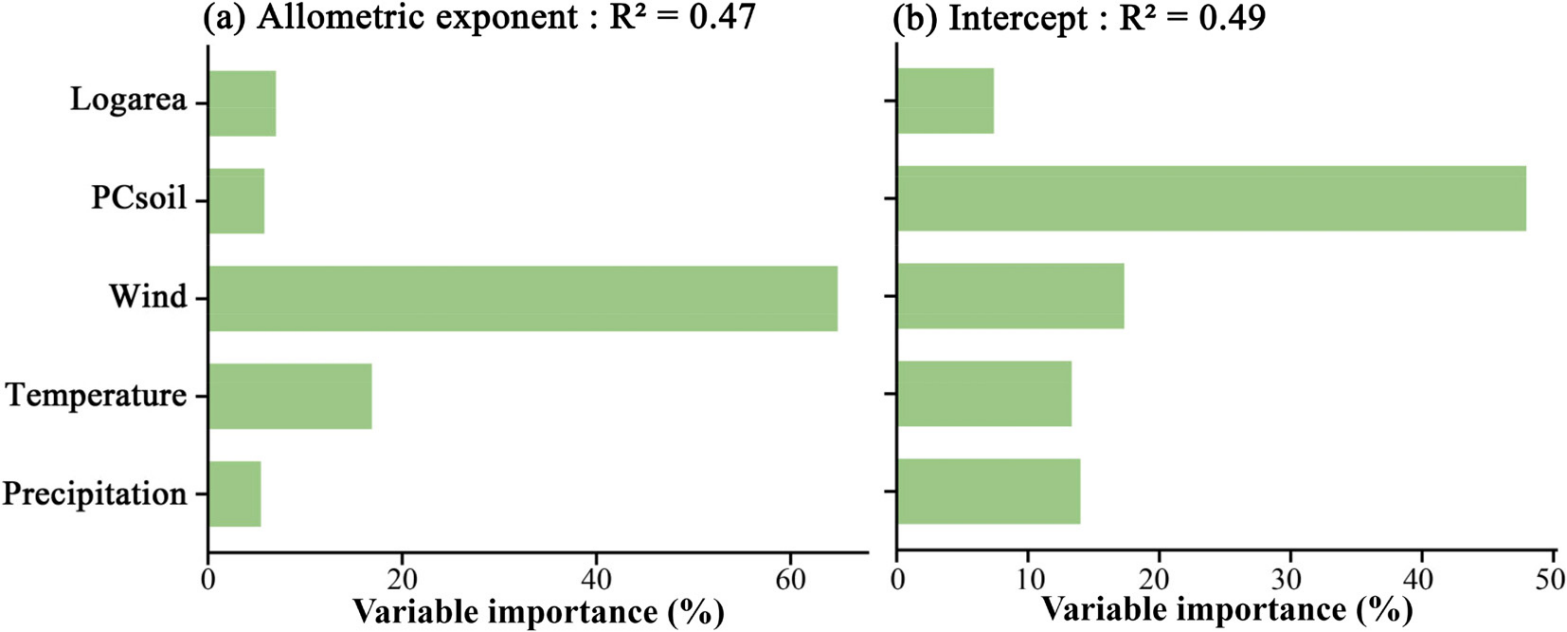
Relative importance of island area, soil properties, and climate factors (wind speed, temperature, and precipitation) in explaining the variation in the height-diameter allometric exponent **(a)** and intercept **(b)** of island plants.

For the height-diameter allometric exponent of plants, island area, soil, and three climate variables explained approximately 47% of the variation in the height-diameter allometric exponent, and wind speed was the most important predictor, accounting for 64.8% of the variation in the exponent, followed by temperature, which accounted for 16.9%. The relative importance of soil factors, precipitation, and island area was low, with soil contributing 5.8%, precipitation 5.5%, and area 7% (**Figure 4a**). For the height-diameter allometric intercept of plants, the five variables explained approximately 49% of the variation in the intercept. Soil was the most important predictor, contributing 47.9% of the variation, followed by wind speed, which accounted for 17.3%. Temperature and precipitation contributed 13.3% and 14.2%, respectively, while area had the least explanation of just 7.3% (**Figure 4b**).

## 4 Discussion

The height-diameter allometric exponent and intercept reflect differences in growth strategies under varying environmental conditions, highlighting the plant adaptability to resource limitations and climate change. This study was conducted to investigate the key drivers of plant height-diameter allometric relationships in island ecosystems, mainly focusing on how island area, soil properties, and climate variables (such as wind speed, temperature, and precipitation) influence plant growth strategies. The results showed that island area had no statistically significant effect on plant height-diameter allometric relationship, while soil factors exhibited a significant influence on the intercept. Among the climate factors, wind speed significantly affected the height-diameter allometric exponent, whereas temperature and precipitation showed no significant effects. Therefore, soil and wind speed play more critical roles in regulating the growth patterns of island plants, while the influences of island area, temperature, and precipitation may be more indirect or limited within the study area.

Island area is generally considered an important factor influencing species richness and various ecological processes (MacArthur and Wilson, 1967; Whittaker et al., 2017; Zhou et al., 2024). However, in this study, we found that the island area did not have a significant effect on the plant height-diameter allometric relationship. Although some studies have shown that island area would affect plant functional traits (Whittaker et al., 2014). We suggest that, although the direct effects are not significant, on larger islands, island area may indirectly influence ecological factors through its impact on soil, temperature, wind speed, and water vapor pressure. Larger islands typically feature more complex topography, better habitat quality, and fewer edge effects (MacArthur and Wilson, 1967; Patiño et al., 2017; Whittaker et al., 2017; Zhang et al, 2021; Zhou et al., 2024). For instance, studies have shown that trees in sheltered valleys are taller than those exposed to strong winds (Thomas et al., 2015), Future research could explore this indirect effect further on larger, more environmentally complex islands.

The influence of soil properties on the height-diameter allometric intercept reached a significant level (*P* = 0.029), indicating that soil factors play an important role in regulating plant growth strategies. Specifically, soil characteristics such as nutrient content, pH, and organic carbon content may shape the height-diameter allometric relationships by influencing plant resource allocation and growth efficiency. Indeed, these soil properties have been shown to significantly affect plant growth rates and patterns across various ecosystems (Cramer, 2012; Givnish et al., 2014; Cysneiros et al., 2021; Xu et al., 2022). For instance, a previous study in the Brazilian forest ecosystem found that soil nutrients were one of the key predictors of tree height-diameter allometric relationship, with nutrient-rich forests supporting taller trees (Cysneiros et al., 2021). Additionally, the physical and chemical properties of soil may further regulate plant growth strategies by influencing the availability of water and nutrients (Li and Fang, 2016). Therefore, the impact of soil properties on plant height-diameter allometric relationship in island ecosystems cannot be overlooked, particularly in resource-limited environments where the role of soil may be even more pronounced.

In this study, climatic factors (i.e., wind speed, temperature, and precipitation) exhibited complex and significantly varying effects on the height-diameter allometric relationship of island plants. Specifically, unlike previous studies (Wang et al., 2006; Zhang et al., 2019), our findings showed that temperature and precipitation had no significant effects on either the height-diameter allometric exponent or the intercept of plants. Generally, rising temperatures accelerate plant metabolic processes and promote growth rates, potentially influencing height-diameter allometric relationships (Zhang et al., 2019; Wang et al., 2006; Hulshof et al., 2015). However, in the study area, variations in temperature did not significantly alter plant height-diameter allometric patterns, which may be related to the fact that the study region is a tropical oceanic island with a relatively narrow range of temperature fluctuations, insufficient to reach the threshold for significantly affecting plant height-diameter allometric relationship. Similarly, precipitation, a key factor influencing plant growth, typically plays a critical role in determining plant growth status by providing water (Hulshof et al., 2015; Cysneiros et al., 2021; Liu et al., 2021). However, in this study, precipitation also showed no significant effect, likely because the islands not only receive water through precipitation but also indirectly supplement moisture from the ocean (Gimeno et al., 2020), thereby mitigating the direct impact of precipitation on plant height-diameter allometric relationship. Nevertheless, under extreme temperature conditions, temperature may still play a crucial role in plant growth, such as extreme high temperatures potentially inhibiting metabolic activities or extreme low temperatures limiting growth rates. Similarly, under extreme precipitation conditions, precipitation may remain a critical factor for plant growth (Cysneiros et al., 2021; Seleiman et al., 2021).

Notably, wind speed exerted a significant negative effect on the height-diameter allometric exponent (**Figure 3**), indicating a remarkably inhibitory effect on the growth patterns of island plants. This finding is partially consistent with the results of Thomas et al. (2015), where plants exposed to higher wind speeds exhibited lower height-diameter allometric exponents. On the one hand, high wind speeds cause mechanical disturbance, directly impacting plants through mechanical damage, leading to sustained mechanical stress and canopy restriction (King, 1986; Meng et al., 2006; Niklas and Spatz, 2006; Jackson et al., 2021);. Many studies have shown that wind speed is one of the main factors limiting canopy height, which in turn inhibits vertical growth (Takashima et al., 2009; Thomas et al., 2015). On the other hand, under high wind conditions, prolonged exposure to wind may drive plants to develop more wind-resistant traits, such as deeper root systems, tougher wood structures, and the formation of more branched growth forms rather than a single trunk (De Langre, 2008; Gardiner et al., 2016). These adaptive changes lead to a growth pattern that favors horizontal expansion rather than vertical growth, further reducing the height-diameter allometric exponent (Telewski, 2012; Gardiner et al., 2016).Although the effect of wind speed on the intercept was not statistically significant in this study, its significant impact on the height-diameter allometric exponent still suggests that wind speed is one of the key environmental factors regulating the growth strategies of island plants.

Based on the relative importance results, different factors exhibited distinct effects on the height-diameter allometric exponent and intercept of island plant height-diameter relationships. Specifically, wind speed among climatic factors and soil properties showed the highest explanatory power for the height-diameter allometric exponent and intercept, respectively. Although numerous studies have demonstrated that island area can directly or indirectly influence island plant communities (MacArthur and Wilson, 1967; Whittaker et al., 2017; Cysneiros et al., 2021; Zhou et al., 2024), its impact in this study was relatively weak. In contrast, among climatic factors, wind speed was the most significant predictor of the height-diameter allometric exponent (contributing 64.8% of the variation), indicating its dominant role in shaping plant growth strategies through mechanical stress and canopy limitation (King, 1986; Niklas and Spatz, 2006; Takashima et al., 2009). On the other hand, soil properties had the strongest influence on the intercept (contributing 47.9% of the variation), reflecting their critical role in determining baseline growth conditions, such as resource allocation and initial morphological adaptation (Cramer, 2012; Cysneiros et al., 2021; Xu et al., 2022). The effects of temperature and precipitation were relatively mild (contributing 16.9% and 5.5% to the exponent, and 13.3% and 14.2% to the intercept, respectively), likely due to the buffering effects of the oceanic climate and moisture supplementation (Gimeno et al., 2020). These results suggest that wind-driven physical constraints (e.g., mechanical damage, canopy limitation, and structural adaptation) primarily regulate the proportional relationship between plant height and diameter (allometric exponent), while soil-mediated resource availability (e.g., nutrient content, pH, and organic matter) predominantly shapes the baseline growth trajectory (intercept). This mechanistic decoupling reveals a multi-dimensional regulatory pattern of plant height-diameter allometric relationship in island ecosystems: wind speed directly influences plant morphological development through external mechanical pressures, forcing plants to tradeoff between vertical growth and horizontal expansion, while soil regulates the starting point and early developmental strategies of plants through resource allocation and initial growth conditions. This distinction not only emphasizes the multi-layered influence of environmental factors on plant growth strategies but also provides new insights into the adaptive evolution of island plants under resource limitations and climatic pressures. Future research could further explore the spatiotemporal dynamics of these driving mechanisms, particularly how the synergistic or antagonistic effects of wind speed and soil resources influence the long-term adaptability of island plants in the context of global climate change.

## 5 Conclusion

Overall, this study reveals the critical roles of wind speed among climatic factors and soil properties in shaping the height-diameter allometric patterns of island plants in the South China Sea region. Wind speed emerged as the primary driver influencing the height-diameter allometric exponent, while soil properties predominantly governed changes in the height-diameter allometric intercept. In contrast, the effects of island area and other climatic factors, such as temperature and precipitation, were relatively weak. These findings uncover the multi-dimensional adaptive strategies of island plants under resource limitations and climatic pressures: wind speed directly shapes plant morphological development through external mechanical stress, whereas soil regulates the starting point of plant growth via resource allocation. The results provide new insights into the driving mechanisms of plant height-diameter allometric relationship in island ecosystems, particularly in the context of global climate change, where the synergistic or antagonistic effects of wind speed and soil resources may influence the long-term adaptability of island plants. These discoveries not only deepen our understanding of plant growth strategies in island ecosystems but also offer important scientific evidence for island ecological conservation and restoration.

## Data Availability

The raw data supporting the conclusions of this article will be made available by the authors, without undue reservation.
